# Metal-free catalysis based on nitrogen-doped carbon nanomaterials: a photoelectron spectroscopy point of view

**DOI:** 10.3762/bjnano.9.191

**Published:** 2018-07-18

**Authors:** Mattia Scardamaglia, Carla Bittencourt

**Affiliations:** 1 Chemistry of Plasma Surface Interactions (ChIPS), University of Mons, Belgium

**Keywords:** catalysis, carbon nanotubes, graphene, metal-free, nitrogen doping, photoelectron spectroscopy

## Abstract

In this review, we discuss the use of doped carbon nanomaterials in catalysis, a subject that is currently intensively studied. The availability of carbon nanotubes since the 1990’s and of graphene ten years later prompted the development of novel nanotechnologies. We review this topic linking fundamental surface science to the field of catalysis giving a timely picture of the state of the art. The main scientific questions that material scientists have addressed in the last decades are described, in particular the enduring debate on the role of the different nitrogen functionalities in the catalytic activity of nitrogen-doped carbon nanotubes and graphene.

## Introduction

Catalytic processes are the basis of many important technologies in the chemical industry and for energy generation, especially in the context of renewable, clean and sustainable energy production [1]. The oxygen reduction reaction (ORR) and the electrocatalytic splitting of water to molecular hydrogen via hydrogen and oxygen evolution reaction (HER and OER, respectively) are fundamental working mechanisms at the cathode of fuel cells, metal–air batteries and dye-sensitized solar cells [2]. However, the current working catalysts are based on expensive metals, such as platinum or its alloys, or metal oxides, which affect the engineering cost of fuel cells being also energy consuming and not highly selective [3]. Therefore, research efforts have been devoted towards alternative highly active catalysts from non-precious metals [4]. Repeatedly reported potential candidates are the less expensive transition metals (Co, Ni, Fe, Mo) and their alloys [5]. However, these metals suffer from inherent corrosion and susceptibility to oxidation which limit their use in acidic electrolytic environments. The most common poisoning agent is CO, which inhibits the dissociative absorption of H_2_ on the catalyst lowering the cell performance [6].

In parallel, the research on the catalytic activity of low-cost and metal-free catalysts has proceeded for decades. The discovery of catalytic properties of carbon alloys with nitrogen dates back to 1926 when Rideal and Wright reported their studies on the oxidation of oxalic acid on charcoal containing nitrogen and iron [7]. In 1966, activated carbon, heated at high temperatures in the presence of ammonia, was used in the cathode of a fuel cell showing an enhanced activity for the electrochemical reduction of oxygen [8]. However, a metal-free catalyst for the ORR had not been considered feasible [9–10] until two fundamental milestones had risen the interest on carbon as an effective replacement of Pt for catalysis. The first one was the prediction of the remarkable electrical conducting properties of carbon nanotubes (CNTs) in 1993 [11–12], rather than their observation, which possibly should be dated back [13]. The second one was the report on exfoliation and characterization of graphene by Geim and Novoselov in 2004 [14]. The impact of these reports on the fundamental research on carbon nanomaterials triggered theoretical and experimental studies exponentially increasing in number in many application fields, including in catalysis [4,15–17].

We present in this introduction the development of the research on carbon nanomaterials in catalysis. The development follows the availability of new carbon nanomaterials and the discovery of their catalytic performance in the ORR: beginning with nitrogen-doped carbon fibres (2006 [18]), followed by carbon nanotubes (2009 [19]) and finally graphene (2010 [20]).

**Figure 1:**
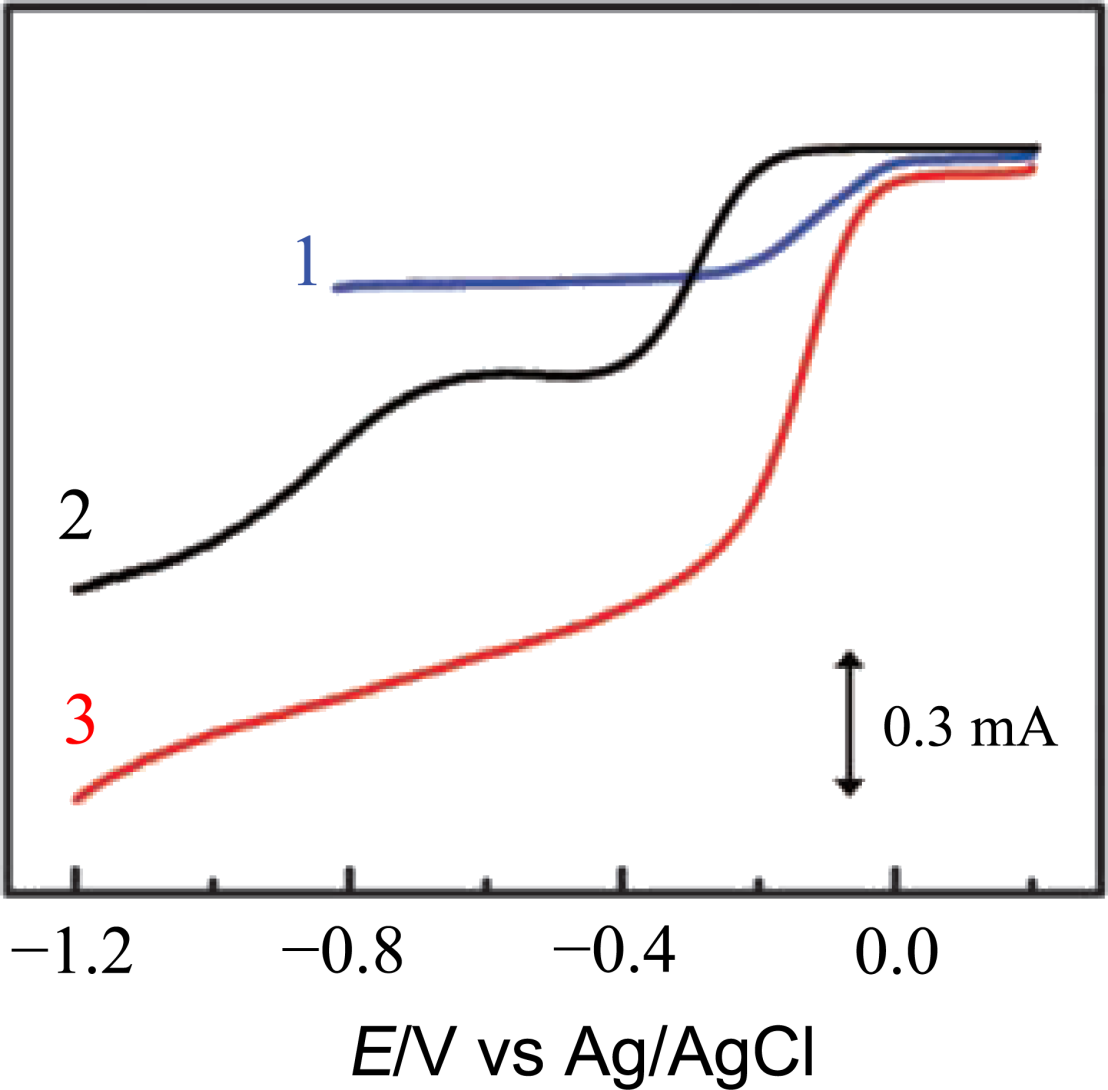
RRDE voltammograms for oxygen reduction in air-saturated 0.1 M KOH at the Pt–C/GC (curve 1), VA-CCNT/GC (curve 2), and VA-NCNT (curve 3) electrodes. Reprinted with permission from [19], copyright 2009 American Association for the Advancement of Science.

In 2006, Matter and Ozkan reported on a metal-free ORR catalyst containing nitrogen-doped carbon fibers. The authors compared the ORR activity of the fibers grown with and without iron. The latter showed significant ORR activity, although they were less performant than the iron-containing catalyst [18]. The first metal-free catalyst that showed an ORR activity superior to commercial Pt in alkaline fuel cells was reported in 2009 by Gong and co-workers [19]; it was based on nitrogen-doped vertically aligned carbon nanotubes (N-vCNTs). Using a rotating ring-disk electrode (RRDE) when measuring steady-state voltammograms in alkaline solution, the authors showed that the ORR activity of N-vCTNs was superior to undoped vCNTs, to randomly oriented N-CNTs, and, in particular, to commercially available platinum-loaded carbon (Vulcan XC-72R), as reported in Figure 1. The electrochemical mechanism for the ORR was the same for aligned and disordered CNTs. The improved electrocatalytic performance of vCNTs was associated with a better-defined surface area of the aligned tips at the interface with the electrolyte solution, which facilitates the electrolyte/reactant diffusion. A strong enhancement of the currents was observed when comparing N-CNTs to undoped nanotubes. Furthermore, they demonstrated that the glassy N-vCNT electrode was considerable more stable toward the ORR than the platinum-loaded electrode, which showed deterioration after repeated cycles associated to migration and aggregation of the Pt nanoparticles. The electrocatalytic activity of nitrogen-doped carbon nanotubes was also reported to be unaffected by CO poisoning, in contrast to commercial electrodes.

Analyzing the RRDE voltammograms for the ORR in air-saturated 0.1 M KOH, the authors showed that the typical curve of pristine vCNTs has a two-step process with onset potential of about −0.2 V and −0.7 V, corresponding to a two-electron pathway that involves the formation of hydrogen peroxide ions as intermediate [19]. The nitrogen-doped (NCNTs) and the commercial Pt–C electrodes exhibit a one-step process for the ORR with a half-wave potential of −0.1 V associated to a more efficient four-electron pathway that directly produces water as end product by combining oxygen with electrons and protons. Furthermore, Gong et al. showed that the steady-state diffusion current was enhanced for N-vCNTs compared to the Pt–C electrode and could be observed over a larger potential range [19].

**Figure 2:**
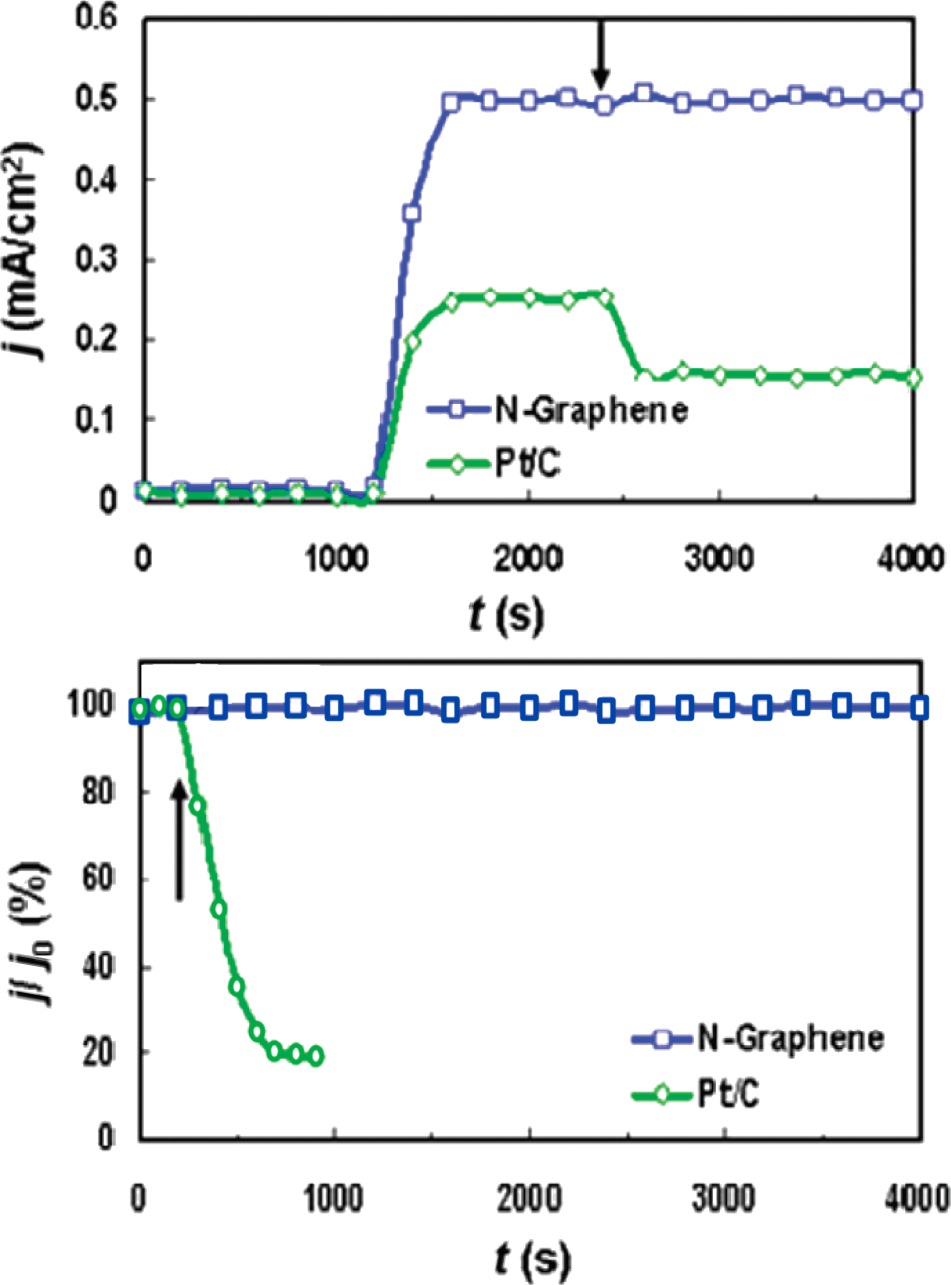
(top) Current density (*j*) as a function of the time (*t*) obtained at the Pt–C (green) and the N-graphene (blue) electrode at −0.4 V in air-saturated 0.1 M KOH. The arrow indicates the addition of 2% (w/w) methanol into the air-saturated electrochemical cell. (bottom) Current (*j*) as a function of the time (*t*) obtained at the Pt–C (green) and the N-graphene (blue). The arrow indicates the addition of 10% (v/v) CO into the air-saturated 0.1 M KOH solution at −0.4 V; *j*_0_ defines the initial current. Adapted with permission from [20], copyright 2010 American Chemical Society.

In 2010, Qu et al. reported for the first time on a nitrogen-doped graphene electrode as replacement for a commercial Pt electrode [20]. Similar to the case of un-doped CNTs [19], the pristine graphene electrode showed a two-step, two-electron process for the ORR, however with onset potentials of −0.45 V and −0.7 V. For N-graphene it is a one-step, four-electron pathway with higher steady-state current with respect to commercial Pt–C electrode. The authors referred to a long-term operation stability and tolerance to poisoning effects, such as the introduction of methanol and CO in the air-saturated electrochemical cell (Figure 2). The response of N-graphene was unaffected by the poisoning, while the Pt–C electrode current abruptly decreased when the gas was introduced.

The three examples reported so far show the performance of nitrogen-doped carbon nanostructures in catalysis and their better efficiency, particularly that of graphene, when compared to commercially available Pt-based electrodes. Nitrogen atoms can be incorporated in the hexagonal carbon lattice in different configurations depending on the number of bonding carbon atoms and to the type of hybridization. The role that the different nitrogen functionalities play in the catalytic mechanism of nitrogen-doped carbon nanostructures is still under intense debate. In this review, we link the fundamental electronic properties to the catalytic performance from a photoelectron spectroscopy point of view. We focus on the discussion of the inherent ORR activity of nitrogen-doped graphene and carbon nanotubes without additional metal catalysts. Although similar in structure, carbon nanotubes (in particular single-walled CNTs) and graphene have different catalytic properties that are mostly due to the curvature of the CNTs. It has been calculated that the reaction energy and the activation barrier of O_2_ molecules on CNTs depends on their curvature and whether the nanotubes are metallic or semiconducting [21]. However, in most of the examples reported in this review concerning CNTs, the CNTs are mostly multiwalled and the theory used in the cited works is usually based on graphene as model system. The most common nitrogen doping techniques and how the doping activates the surface are described, and the spectroscopic differences regarding the nitrogen incorporation in graphene and CNTs are discussed.

## Review

### Heteroatom-doped carbon nanostructures

Carbon nanotubes and graphene share many characteristics in structure and properties, such as high aspect ratio, good mechanical properties and extraordinary electronic properties. The one atom thick two-dimensional planar structure of a graphene sheet makes it superior to CNTs for certain applications because it facilitates the electron transport [22], a key feature of a working electrode. Therefore, graphene may be used in many fields where CNTs have been already used. The excellent mechanical properties, low reactivity and high stability [23] ensure a long-time use as a catalyst under working conditions. Another very important property of graphene is its high thermal and electrical conductivity [24], allowing for a good heat diffusion and conduction during catalytic reactions, preventing the generation of overheated regions.

The Dirac point of pure graphene is found exactly at the Fermi level, hence graphene is defined as a semi-metal (or a zero-gap semiconductor). This peculiarity represents one of the biggest challenges for its use in concrete applications, because it results in a very low density of states (DOS) at the Fermi level for typical doping levels, making graphene an intrinsically inert material. To overcome these issues, several strategies have been employed to tailor the properties of graphene. Being very sensitive to local perturbations, any modification of the lattice or adsorption of foreign atoms or molecules produce sudden evident changes in the density of states that can be monitored by the shift of the Dirac cone or by a change in the resistance [25–28]. To meet the specific requirements of an application, chemical modification of pristine carbon nanomaterials is essential to enable, for example, an improved sensitivity to atoms and molecules interacting with the modified surface of the carbon nanostructure.

The creation of active sites for interaction is a key step for obtaining an optimal working catalyst as well as for sensing foreign species [29–31]. These sites enable the dissociation of molecules and the formation of bonds with the products, for example in the molecular adsorption of oxygen and its reduction. On graphene, a 2D surface without bulk, every atom takes part to the interaction mechanism, providing the highest conceivable specific surface area (above 2600 m^2^·g^−1^) [26] and also a high density of surface active sites. In contrast to many metal catalysts, graphene has no subsurface [32–33], consequently the chemical complexity is confined to the two-dimensional interface between graphene and the reactants, without the possibility of intercalation chemistry.

In addition to the properties discussed above, the easiness of tuning makes nanocarbon materials very adaptable. Indeed, the graphitic network can be easily modified by the introduction of heteroatoms, functional groups or defects, such as dislocations, vacancies or edges [25,34–35]. They act as active sites for electron localization or they are useful to anchor metal clusters or foreign molecules that will, in turn, be active sites. A change of the reactivity can be thus obtained in a controllable way for systematic optimization. Changing the structure of current metal catalysts in order to study their dynamic behavior is a difficult task and rarely investigated [36]. In contrast, this is routinely done with graphene and carbon nanotubes.

**Figure 3:**
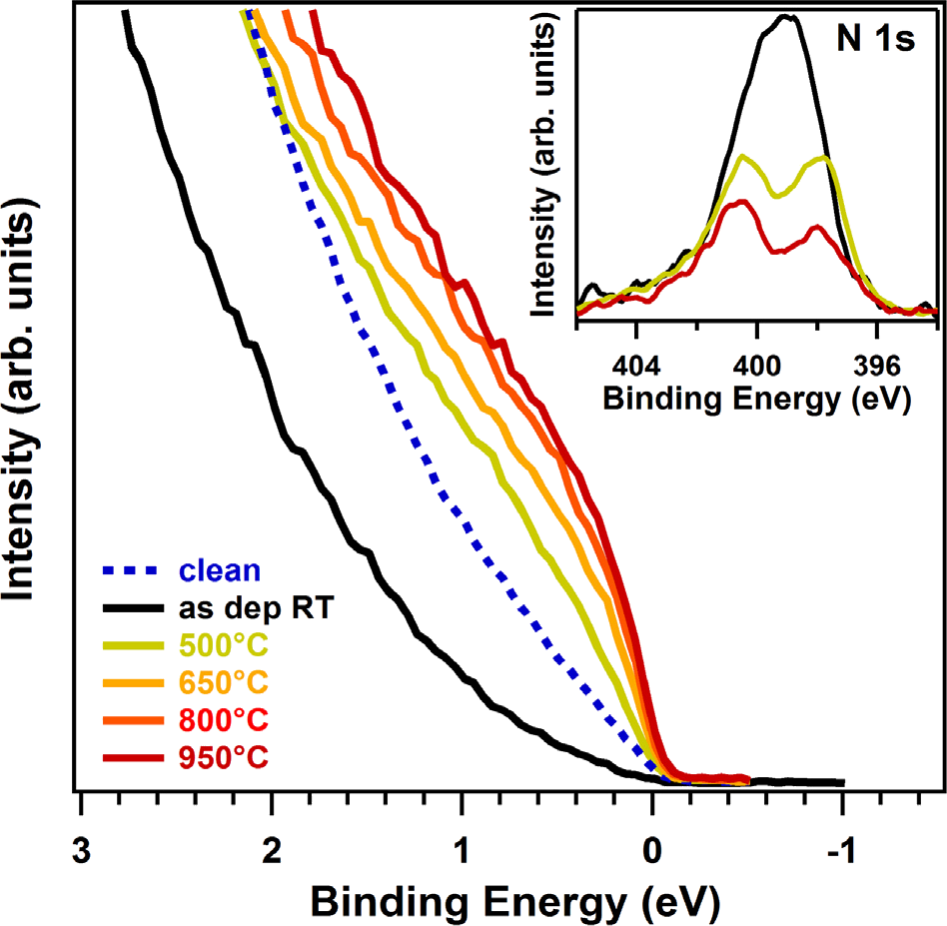
Valence band spectra near the Fermi energy level for pristine, nitrogen functionalized (“as dep RT”) and annealed nitrogen-functionalized v-CNTs. The integrated spectra were recorded normal to the CNT tips, using a photon excitation of 31 eV. In the inset are reported the XPS N 1s core levels recorded after the nitrogen functionalization (black) and after two heating treatments: 500 °C (yellow) and 950 °C (red). Reprinted with permission from [58], copyright 2014 Elsevier.

Among the strategies adopted to dope graphene, heteroatom doping is a promising way to improve its reactivity by introducing active sites that facilitate the interaction with foreign gases already at room temperature [29]. Heteroatoms change the electronic properties of graphene, modify its morphology and, in addition, activate its surface. Heteroatom, or substitutional, doping is obtained when a foreign atom replaces a carbon atom in the hexagonal lattice; typical heteroatoms are nitrogen [37–41], boron [42–44], sulfur [39,45–46], and phosphorous [47–49]. Nitrogen and boron have a similar atomic radius as the carbon atom. The number of electrons differs by one (seven in N, five in B, hence a different electronegativity), which makes N and B straightforward models to achieve n- and p-type doping of graphene, respectively. Similarly to nitrogen, phosphorus has five valence electrons, but its atomic radius is bigger. Therefore, its incorporation in the carbon lattice causes a protrusion out of the graphitic plane [47]. Other functional groups containing oxygen [50], hydrogen [51–52] or fluorine [53–55], also contribute to the modification of the electronic states of graphene. In general, point defects generate localized states at the Fermi level, easily identifiable as protrusion by scanning tunneling microscopy (STM) [56], while carbon vacancies are responsible for an opening of the energy gap [57]. The increase in the density of states around the Fermi level after doping [58] usually leads to an enhancement of the catalytic activity of the material. In the particular case of nitrogen doping, the nearest-neighbor carbon atom of a nitrogen atom is considered to be the active site [19,26] because of the net positive charge accumulating on it due to the higher electronegativity of the N atom [59–60]. Thus, it will attract electrons from the anode more easily and facilitate the ORR. Yu et al. [59] using DFT calculations showed the increase of the density of states at the Fermi level of the C atom neighbor of a N atom. This is due to the combination of two effects: the higher electronegativity of nitrogen and the back-donation of lone-pair electrons from N to C. In this context, Scardamaglia et al. evaluated the increase in the DOS at the Fermi level of nitrogen-doped vertically aligned carbon nanotubes (N-vCNTs) by measuring valence band (VB) spectra with high energy resolution [58]. They correlated the change in the DOS to the different types of nitrogen functionalities incorporated in the carbon matrix, using X-ray photoemission spectroscopy and in situ annealing. Specifically, it was shown that through annealing at temperatures up to 950 °C the amount of pure substitutional nitrogen (graphitic) and the DOS increase (Figure 3). As mentioned, the low-energy electronic excitation and the selectivity for oxygen dissociation strongly depend on the type of nitrogen dopant present in the sample, as we will discuss in the next paragraph. The increasing interest in new functionalization strategies of carbon nanomaterials have been triggered by the wide range of potential applications.

### Co-doping

After the reports on the catalytic performance of nitrogen-doped carbon nanomaterials for the ORR, the scientific interest turned to the possibility of co-doping the carbon nanomaterials with nitrogen and another element aiming at a further enhancement of those properties through synergistic effects. The first report showing co-doping to enhance the metal-free catalytic activities of carbon-based catalysts dates back to 2011 and it is based on vertically aligned boron–carbon–nitrogen (BCN) nanotubes [61]. Similar to nitrogen, boron can take a substitutional position with three-fold coordination or form a vacancy complex changing the bond lengths in the carbon lattice. These configurations respond differently to a gaseous analyte [29]. Wu et al. explained the interesting interaction between oxygen and boron-containing carbon nanomaterials: because it is an electron acceptor, B easily oxidizes carbon atoms in a site-dependent way [62].

Besides boron [61,63–64], other elements that have been co-doped with nitrogen are sulfur [65] and phosphorus [66]. In 2015, Zhang and co-workers synthesized a carbon foam co-doped with nitrogen and phosphorous, which was the first bifunctional electrocatalyst for both ORR and OER [49] for high-performance rechargeable zinc-air batteries. The same material was used by Xue et al. as a counter electrode in dye-sensitized solar cells [67]. A high-performance anode material for lithium-ion batteries was obtained using graphene co-doped with nitrogen and fluorine, which was prepared by a hydrothermal reaction of an aqueous dispersion of graphene oxide with trimethylamine trihydrofluoride [68].

**Figure 4:**
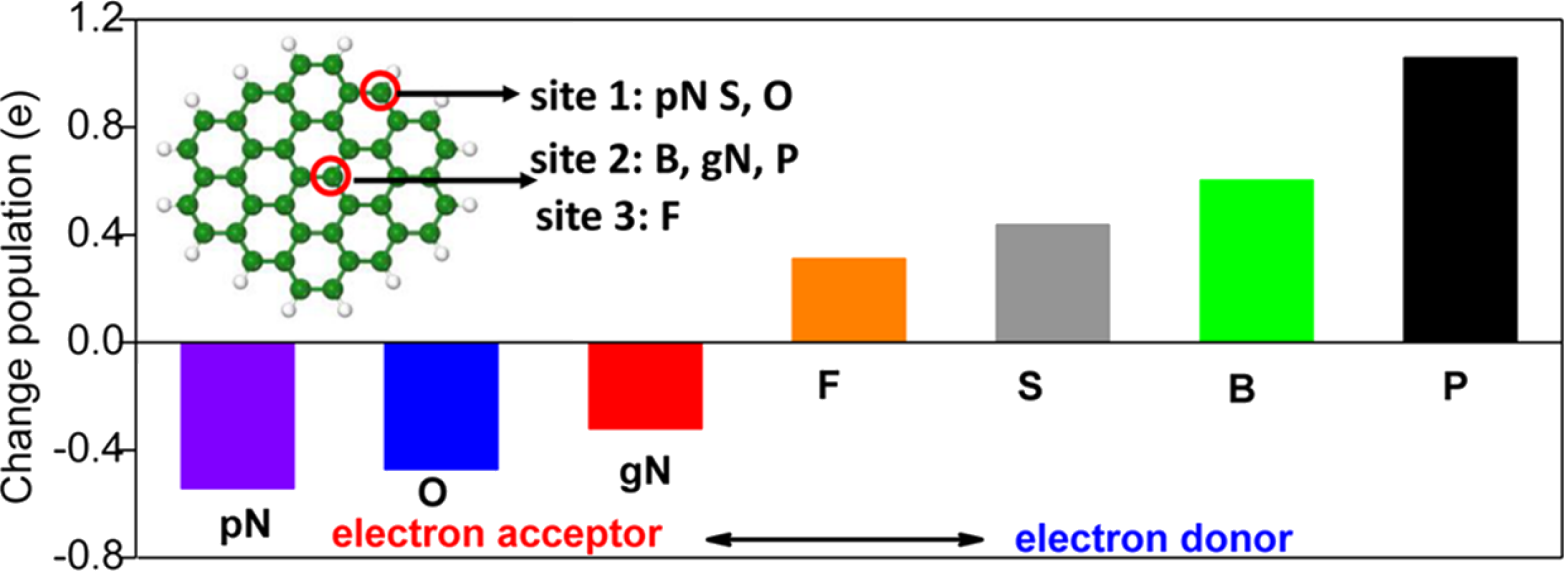
Natural bond orbital (NBO) population analysis of six different non-metallic heteroatoms in a graphene matrix. pN and gN represent the pyridinic and graphitic configurations of N, respectively. The inset shows the proposed doping sites for different elements. Sites 1 and 2 are the edge and center in-plane sites, respectively. Site 3 is an out-of-plane center site in graphene. Reproduced from [66], copyright 2014 American Chemical Society, published under a CC-BY 4.0 International license, https://creativecommons.org/licenses/by/4.0/.

In nitrogen-doped materials, dual doping promotes long-term stability and resistance to poisoning agents for working catalysts. In general, the principle of dual doping is the introduction in the hexagonal carbon lattice of two elements with reverse electronegativity with respect to that of carbon (χ = 2.55), for example, N (χ = 3.04) and B, P or S (χ = 2.04, 2.19, 2.58, respectively), as illustrated in Figure 4. The coupling of the donor and acceptor activity of both dopants generates an increase in the ORR activity, with better performance than single-doped materials. The reason for the enhanced catalytic activity cannot be simply associated to an increase in the available active sites. In addition, synergistic effects of both dopants need to be taken into account.

### Synthesis and properties of nitrogen-doped graphene

Nitrogen doping of carbon nanostructures can be performed either during the synthesis or by post-synthetic treatments. The most common technique for doping during the synthesis is chemical vapor deposition (CVD), similar to the synthesis of the pristine material [36,69–70], albeit using nitrogen-containing precursors such as benzylamine [71], acetonitrile [72–73], phthalocyanines [74–75] or ammonia [76]. Post-synthetic treatments are more effective to achieve surface functionalization, while the use of nitrogen-containing precursors along with a carbon source is more suitable to obtain a homogeneous incorporation of nitrogen in 3D graphene foam [77], multiwalled CNTs [78] or porous carbon [79].

**Figure 5:**
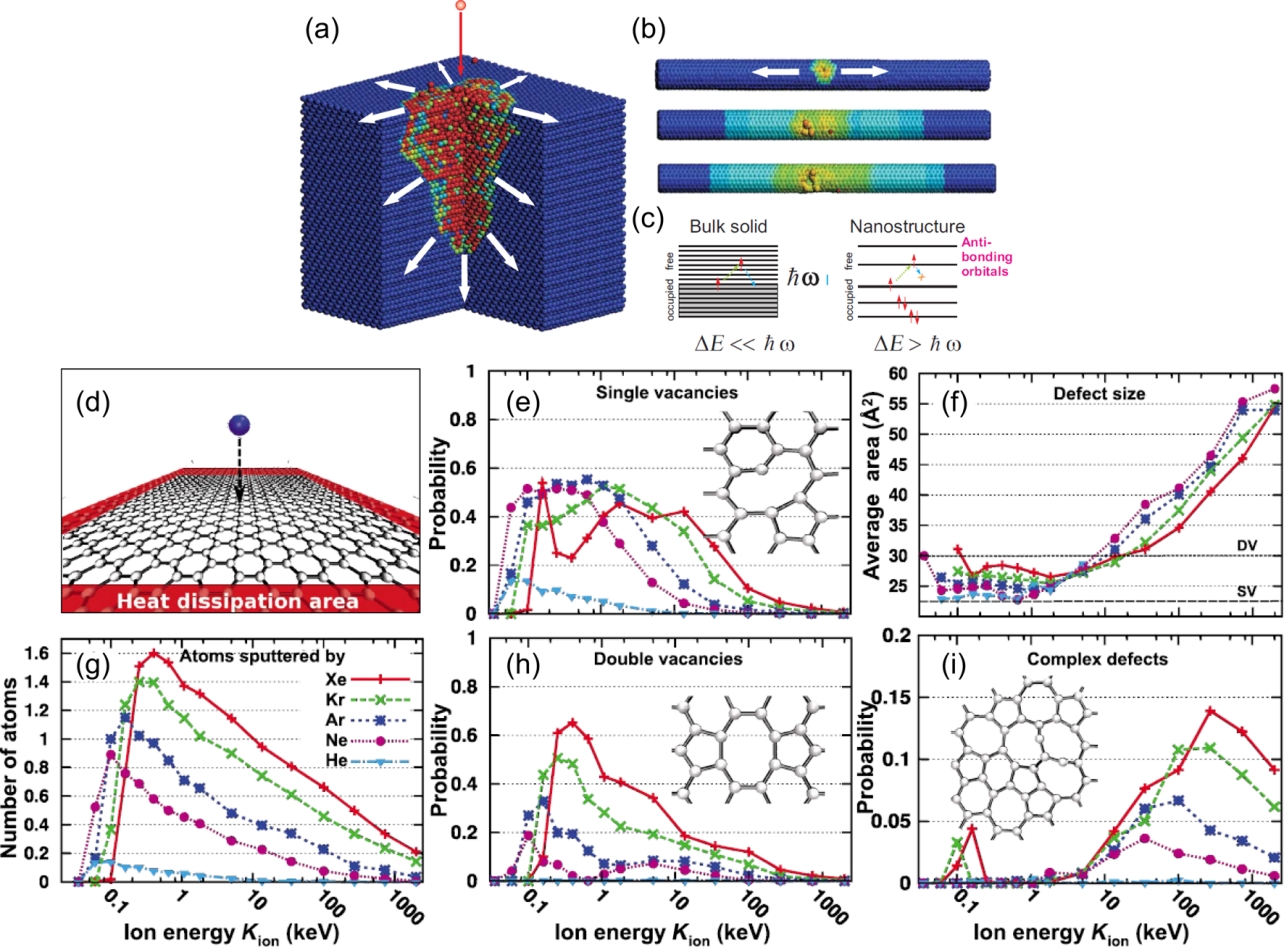
Figure 5 (a–c) Conversion of the projectile initial kinetic energy into thermal energy in bulk and nanosystems: (a) impact of an energetic ion onto a bulk metal target. The ion kinetic energy is transferred ballistically to the target atoms, which results in a temperature raise. The excess energy is dissipated in an essentially 3D system. The atoms are colored according to their kinetic energy from blue (zero energy) to high (red) energies. A quarter of the target was cut out for a better visualization. (b) Impact of an ion onto a carbon nanotube, a quasi-1D system. The excess energy is dissipated in only two directions, which may affect the temperature profile and give rise to additional defects. (c) The sketch of the electronic structure of bulk and nanoscale objects, illustrating the so-called “phonon bottleneck” phenomenon. The excitation relaxation time is enhanced when the spacing between the size-quantized energy levels Δ*E* is larger than the vibrational energy ħω. This mechanism is discussed for illustration purposes only. There are many other nonradiative relaxation channels in nanosystems that affect the excitation lifetimes. Reproduced with permission from [83], copyright 2010 American Institute of Physics. (d–i) Production of defects in graphene under ion irradiation as revealed by the analytical potential molecular dynamics: (d) simulation setup. (e, h) Probability for the formation of single and double vacancies as a function of the ion energy. The insets show the atomic structures of the reconstructed vacancies. (f) Average area of defects as a function of the ion energy. The areas corresponding to single vacancies (SV) and double vacancies (DV) are marked. (g) Number of sputtered atoms per ion impact as a function of the ion energy. (i) Probability for creating defects other than SV/DV (except Frenkel pairs or Stone–Wales defecs), see the inset for an example. Reprinted figure with permission from [86], copyright 2010 American Physical Society.

Post-synthesis treatments such as cold plasmas and ion implantation emerge as versatile options to engineer nanostructured carbon materials with different elements [80]. These techniques allow for high dopant concentrations (about 10 atom %) and for the design of different architectures adjusting the ion kinetic energy or plasma parameters as well as the annealing conditions [81–82]. Recently, in accordance with the theoretical report of Krasheninnikov et al. [83], Scardamaglia et al. showed that for the doping of carbon nanostructures in a suspended geometry, the use of energetic ions with kinetic energies from hundreds of electronvolts to few kiloelectronvolts does not lead to a damage or the sputtering of the material [37,57,84–85]. This effect was explained with different dissipation of the ion energy in the nanostructured material compared to the bulk. In the former, many ions traverse the nanostructures without inelastic scattering, while for bulk materials or graphene supported on heavy metallic substrates, the energy of the incident ion species is dissipated at the surface generating a backscattering cascade leading to the sputtering of the sample surface (Figure 5). Using molecular dynamics combined with analytical potential and density functional theory methods, Lehtinen et al. reported on the influence of the ion kinetic energy and mass on the probability of defect formation during irradiation of suspended graphene sheets and single-walled CNTs [86]. Being proportional to the ion mass, the aforementioned probability is lower for nitrogen than for argon. The energy required to displace a C atom from the hexagonal network was reported to be about 22 eV [87]. This allows for the creation of carbon vacancies that are subsequently filled by N atoms. It was shown that at higher kinetic energies, the cross section for vacancy creation decreases and more complex defect configurations are created, such as di-vacancies and distortions (Figure 5) [83,86–87].

The effect of different ion kinetic energies on supported graphene has been highlighted in the case of CF_4_ plasma: for energies close to the threshold for carbon substitution, functionalization takes place, while at higher energies the main electronic signatures of graphene are compromised indicating the destruction of the hexagonal lattice due to the metallic atoms backscattering from the substrate [55,88]. In contrast, for suspended graphene, using similar ion kinetic energies, the type of defects was mainly affected while maintaining a clean and non-destructive functionalization [37,55,89]. Theoretical reports showed also that if the ion energy is set slightly below the threshold needed to create single vacancies, a higher substitution-to-defect ratio can be reached [87].

**Figure 6:**
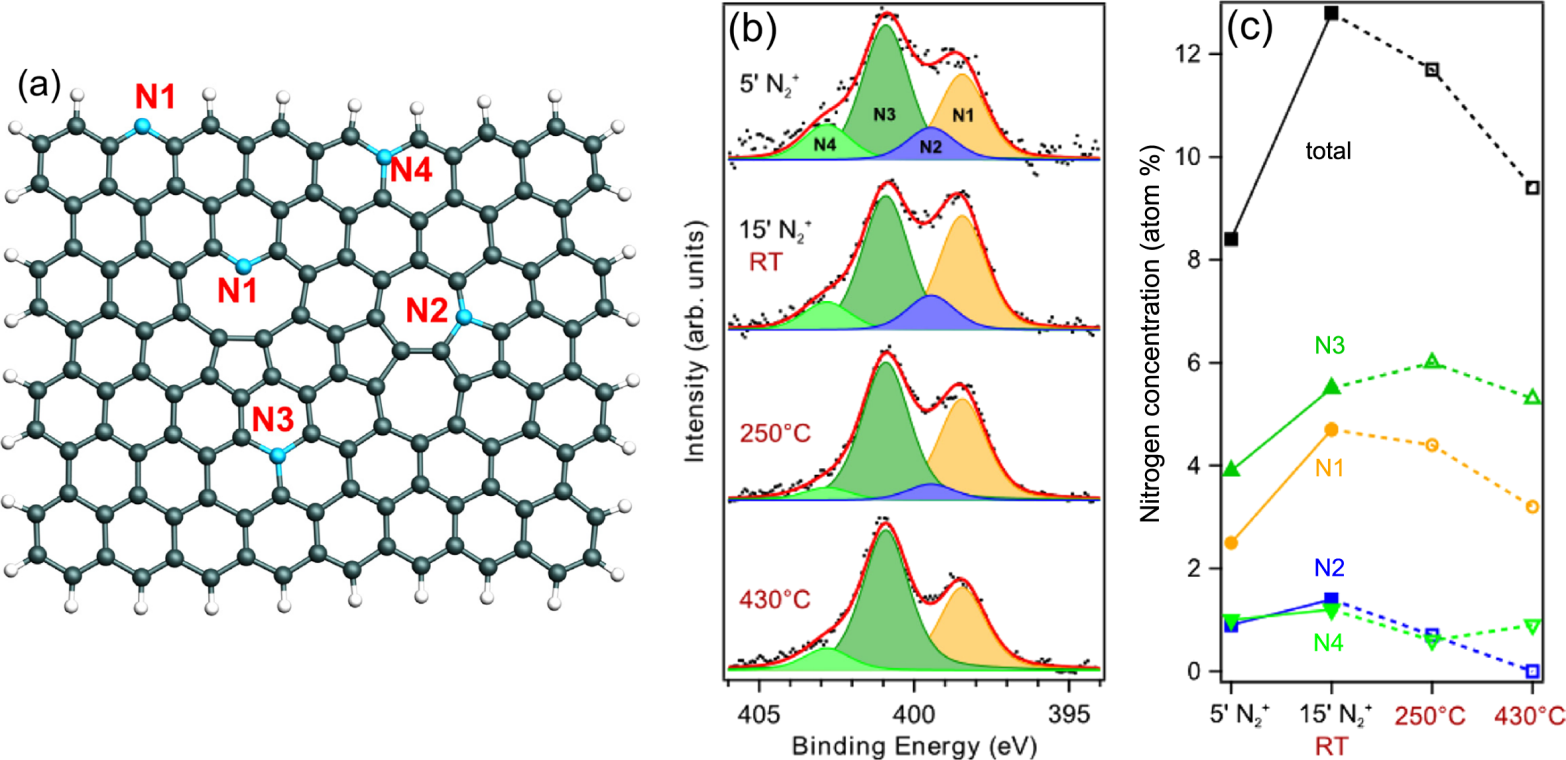
(a) Schematic representation of nitrogen configurations in graphene. Nitrogen atoms given in blue: (N1) pyridinic N, (N2) pyrrolic N, (N3) graphitic N, and (N4) graphitic ‘‘valley’’ N. These are only examples of structures and do not represent an exhaustive list of possibilities. (b) N 1s core level spectra corresponding to 5 and 15 min nitrogen implantation of CVD few-layer graphene at 250 °C and 430 °C annealing from top to bottom, respectively; experimental data (dotted line), peaks resulting from a least-square fitting procedure (continuous red line). A Shirley-type background was subtracted. (c) Nitrogen content in the sample for each configuration and total amount (black squares) as a function of the implantation time (full symbols connected by full line) and of the annealing temperature (open symbols connected by dotted line). The second and third points are connected even though they are two different studies: nitrogen concentration after 15 min of implantation (second point) and after annealing to 250 °C (third point). Adapted with permission from [37], copyright 2014 Elsevier.

Other advantages of the post-synthesis functionalization methods include the possibility to perform the doping after the integration of the nanostructures in a device and the possibility to create different nanopatterns using appropriate masks [90]. In this context, plasma-based methods (radio frequency or microwave) have been proved to be effective techniques to graft different atomic species, such as oxygen [82,91], fluorine [54–55], nitrogen [57,92–93] and boron [94], on both carbon nanotubes and graphene. However, to avoid contamination with undesired elements, these methods require an UHV environment and typical surface preparation techniques should be considered. Controlled doping using plasma results in a change of the electronic properties without affecting the morphology. In particular, in the case of nitrogen doping, Scardamaglia et al. [37] showed that the sp^2^-character of the material is maintained or easily recovered through thermal annealing (Figure 6). A nitrogen atom can be hosted in the hexagonal carbon network in many forms. The three most common configurations are pyridinic (N1), pyrrolic (N2) and graphitic (N3, N4). While a pyrrolic N atom is in a pentagonal structure, pyridinic and graphitic N atoms are sp^2^-hybridized and, in both configurations, the N atom takes the place of a C atom. It is important to note that the graphitic configuration is purely substitutional while the pyridinic configuration exhibits a vacancy as neighbor.

The different nitrogen configurations are easily distinguished by XPS according to the binding energy of the N 1s core level spectra. The pyridinic configuration is usually found at 398–399 eV, the pyrrolic configuration and other defective components at 399.5–400.5 eV, and the graphitic configuration at 400–401 eV [4,95–96]. At slightly higher binding energies with respect to graphitic N, the component was assigned to graphitic valley N [97], which means a graphitic configuration close to a vacancy or edge (N4 in Figure 6a). Nitrogen oxide is usually found at binding energies higher than 402 eV [98]. Other C–N components, such as single bonds, N adatoms or Stone–Wales defects (three-fold coordinated N atoms comprised in two pairs of five-membered and seven-membered rings), may be found at the position corresponding to pyrrolic nitrogen. Hence, their precise determination is not possible by using only XPS and other techniques need to be used, e.g., infrared spectroscopy and electron microscopy.

**Figure 7:**
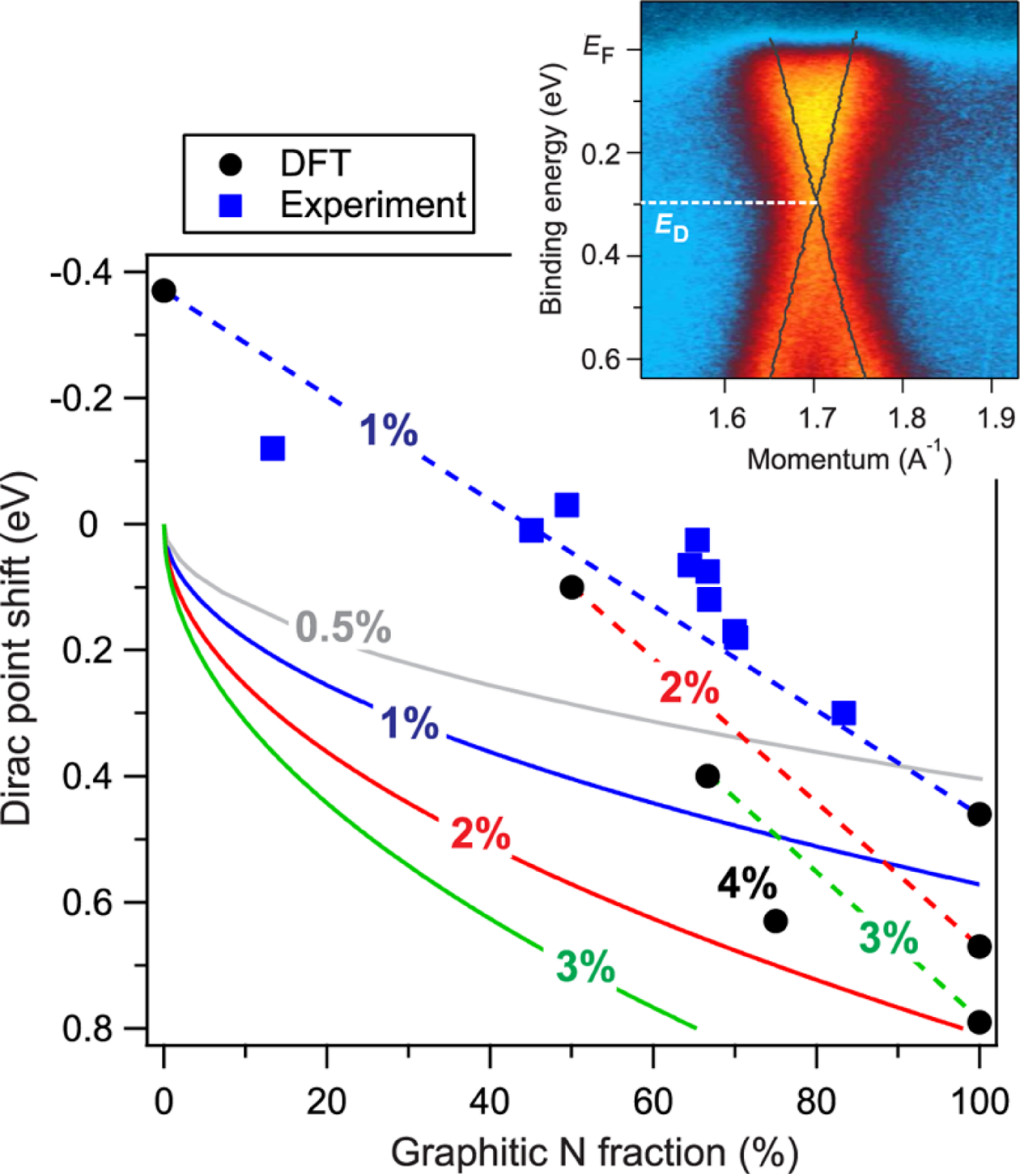
Measured Dirac point position (blue symbols) at different concentrations of pyridinic and graphitic N, compared to the DFT calculation results (black circles connected by dashed lines). The numbers denote the total concentration of nitrogen (NP + NG). Solid lines demonstrate the hypothetical situation when pyridinic N provides no charge transfer while graphitic N donates one electron. The inset shows the ARPES intensity of an N-graphene/Au/Ni(111) system at the K-point of the Brillouin zone, measured after nitrogen conversion in the ΓK direction at a temperature of 40 K. Reprinted with permission from [99], copyright 2014 American Chemical Society.

Content and configuration of nitrogen in graphene and carbon nanotubes can be tuned by thermal annealing or by the interaction with different substrates. Temperature treatments were reported to promote the conversion of pyridinic N into graphitic N and the fine-tuning of the density of states at the Fermi level, which increases proportionally to the content of graphitic N in the carbon lattice. The five valence electrons of the N atoms are distributed differently. Thus, in the graphitic and pyridinic configurations, nitrogen atoms have different effects on the electronic properties of graphene. While the former, three-fold coordinated, shares the additional electron with the graphene network in a partially occupied π*-band, which becomes available for conduction, the latter has only two neighboring carbon atoms to form two σ-bonds. One electron occupies the p*_z_*-orbital and the other two form an electron lone pair without occupying the π*-band. Therefore, pyridinic nitrogen does not behave as an electron dopant. Instead, the pyridinic vacancy complex has a hole-doping effect. This behavior is reflected in the DOS at the Fermi level, and it can be clearly observed in Figure 3 in which the VB spectra of N-doped CNTs are reported together with the respective N 1s core level spectra [58]. After plasma exposure, the amount of pyrrolic and pyridinic nitrogen components was higher than that of graphitic N. This is reflected by a decrease of the DOS with respect to the pristine sample. When UHV annealing was performed, the N 1s line shape of the CNTs (onset of Figure 3) evolved due to the desorption of pyrrolic N and the transformation of pyridinic N into graphitic N. Both effects were reported to be responsible for the increase in the DOS. In graphene, the modification of the DOS is reflected in a shift of the Dirac cone, as confirmed experimentally recently by changing the ratio between graphitic N and pyridinic N through thermal treatments [57] and by the intercalation of metal atoms [99]. The authors reported the calculated and experimental Dirac point positions for different pyridinic/graphitic ratios (Figure 7). There is a counterbalancing effect between the two doping types that obscures the evaluation of the effective charge transfer to/from the dopant atoms.

The nitrogen incorporation in graphene and the consequent changes of the spectroscopic signature are slightly different compared to CNTs. The curvature of the nanotubes (more accentuated for SWNTs) causes a partial rehybridization of the carbon orbitals deviating from a pure planar sp^2^-configuration. Some sp^3^-contributions can be thus detected, also due to presence of pentagons and intrinsic defects on the tips [100]. Under similar doping conditions the amount of pyrrolic nitrogen is therefore higher in CNTs than in graphene [85]. Furthermore, the nitrogen incorporation in graphene is influenced by the substrate that supports the graphene sample. Both the morphology (moiré pattern) and the electronic interaction play a role in the selectivity of preferential N sites [92,99].

The amount of nitrogen is also an important parameter to preserve the stability and the electronic properties of graphene. It has been shown that there is an upper limit for the local concentration of substitutional nitrogen in an sp^2^-layered carbon system [95]. For bilayer graphene, which in good approximation can be extended to MWNTs, the system remains fully sp^2^-coordinated below a nitrogen concentration of 15–20 atom %. Above this threshold cross-linking between the layers starts to occur and an sp^3^-coordination of carbon begins to occur [95]. At intermediate concentrations, the system is stabilized by nitrogen saturation of edges and vacancies, implicating an increasing content of pyridinic N compared to purely substitutional N atoms, as also observed experimentally in ion-bombarded suspended graphene [37].

### The role of the active sites

Nitrogen doping of carbon nanomaterials improves their catalytic activity, which in some case surpasses the performance of commercially available metal-based catalysts. As shown above, nitrogen can be hosted in the hexagonal carbon lattice in many configurations (sites) but, to date, their individual role in the actual catalytic performance is not clear and subject of controversy. In this section we will overview several reports in which this aspect was addressed. The literature on the catalytic performance of nitrogen-doped carbon nanomaterials is vast. However, there are only few research groups that performed detailed X-ray photoelectron spectroscopy measurements on their samples. In the already mentioned work by Qu et al. [20], one of the first reports on the electrocatalytic performance of N-doped graphene, the authors found a threefold steady-state current density with respect to a commercial Pt–C electrode. This was the first report where N-doped graphene was employed as electrode for the ORR with the performance surpassing the commercial electrodes. Although the role of the active sites was not addressed in detail, the electrode performance was remarkable. The N/C ratio was approximately 4 % but the energy resolution of the reported XPS data was too poor to correctly distinguish the different N species. The N 1s peak was deconvoluted into two components: pyridinic N at 398.3 eV and pyrrolic N at 400.5 eV. The absence of the peak of graphitic N may be due to a poor data analysis of the spectra, rather than due to a physical reason.

**Figure 8:**
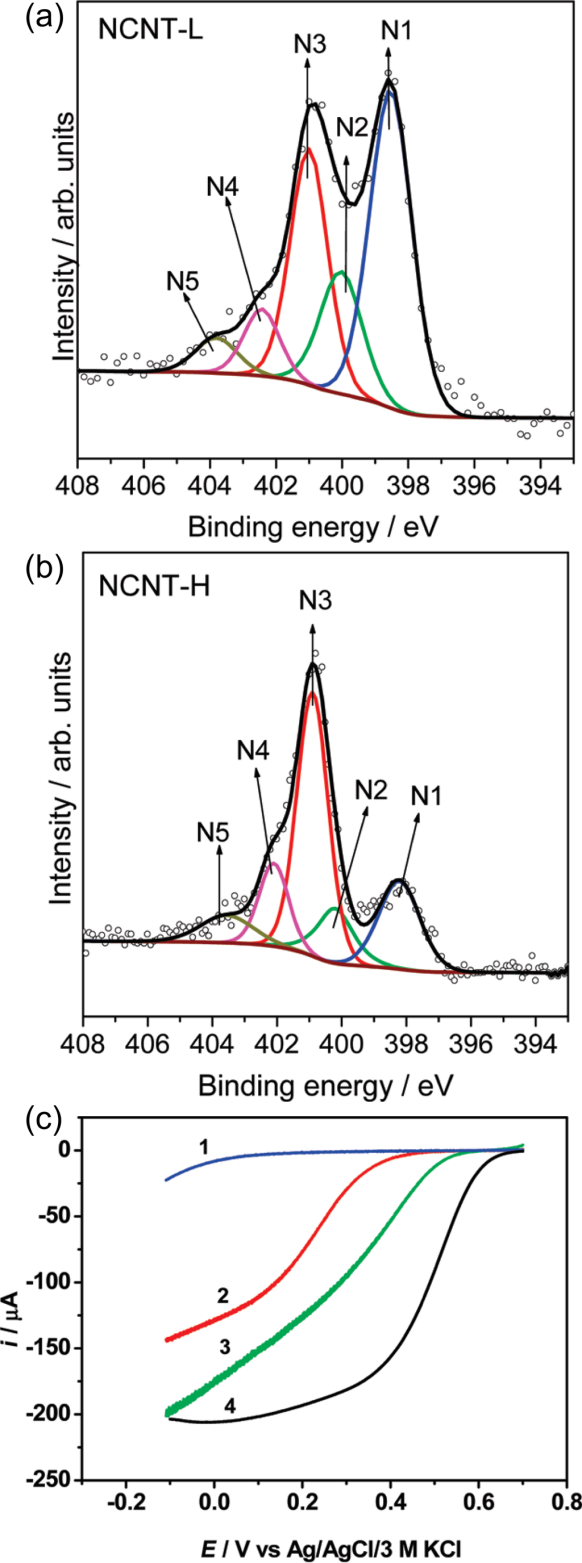
N 1s core level spectra for N-CNT samples grown at (a) 550 °C and (b) 750 °C (N1: pyridinic N, N2: pyrrolic N, N3: graphitic N, N4: pyridine-N-oxide, and N5: chemisorbed nitrogen oxide). (c) RDE linear sweep voltammograms for the ORR in oxygen-saturated 0.5 M H_2_SO_4_ at a rotation speed of 900 rpm and a scan rate of 5 mV/s with (1) un-doped CNT, (2) NCNT-H, (3) NCNT-L, and (4) E-TEK Pt–C (20 wt %) as catalyst. Adapted with permission from [103], copyright 2009 American Chemical Society.

The correlation between catalytic activity and different nitrogen functionalities was investigated by Rao and co-workers [101]. Evaluating the ORR activity of v-CNTs with different amounts of incorporated nitrogen (from 4 to 11 atom %), they found that the v-CNTs with the highest amount of pyridinic N showed the highest onset potential (the potential where the current density increases to 10 µA/cm^2^) and highest positive reduction peak (ca. 0.35 V). The results obtained using nitrogen-doped v-CNTs were superior to those using un-doped nanotubes but less remarkable than those of commercial Pt–C electrodes. Nevertheless, they attributed the improved performance to the conjugation effect of the nitrogen lone-pair electrons found in the π-system of pyridinic N and graphene, as already reported for carbon nanofibers by Maldonado and Stevenson [102]. A more detailed study was performed by Kundu et al. [103] on N-CNTs prepared via pyrolysis of acetonitrile over cobalt catalysts. Samples were synthesized at different temperatures, 550 and 750 °C, allowing one to obtain approximately the same amount of nitrogen (around 7 atom %) but different ratios between graphitic N and pyridinic N of 0.7 and 2.3, respectively. The obtained N 1s core level spectra are shown in Figure 8a,b. The reported catalytic activity measured by CV and RDE in oxygen-saturated H_2_SO_4_ at 900 rpm is shown in Figure 8c. The results indicate that the catalytic activity for the ORR of nitrogen-doped CNTs with an onset potential at about 0.5 V is much higher positive than that of pristine CNT, although the onset potential is still 0.1 V lower than that of the Pt–C catalyst. In particular, the performance of the sample with a higher amount of pyridinic nitrogen (NCNT-L) is superior to the one with a higher amount of graphitic nitrogen (NCNT-H).

The reports of Rao and Kundu mentioned above suggest that in carbon nanotubes pyridinic nitrogen is responsible for the high catalytic performance in the ORR, although they reported a lower efficiency compared to commercial Pt catalysts. Remarkably, Luo and co-workers succeeded to synthesize graphene dope with purely pyridinic N by CVD of hydrogen, ethylene and ammonia on Cu foils [104], with nitrogen contents up to 16 atom %. Graphene with only one type of N dopant is an optimum platform to study the role of this particular site in the ORR activity. However, contrary to the previous reports on CNTs that attributed the high ORR performance to pyridinic N, Luo et al. showed that all the graphene samples had a poor ORR activity with 0.3 V difference in the onset potential compared to a Pt disk. The low performance was associated to the mutual repulsion between the lone-pair electrons in both pyridinic nitrogen and O_2_, which causes a higher energy barrier for the activation of molecular oxygen. The lower catalytic performance of graphene with pyridinic N compared to N-CNTs can be attributed to the intrinsically higher reactivity of the nanotubes due to their curvature [21].

Three other groups reported that catalysts with a higher amount of graphitic N exhibit a higher ORR activity [105–107]: Niwa et al. [105] described carbon nanostructure alloys (a precursor of graphene), Nagaiah and co-workers studied nitrogen-doped CNTs [106], and Parvez et al. used N-graphene [107].

In the first report, correlating X-ray absorption spectroscopy (XAS) and ORR activity, the study was focused on the introduction of nitrogen into various carbon-based cathode catalysts for the polymer electrolyte fuel cell (PEFC) [105]. Different preparation methods were used: nitrogen doping using ammonia resulted in high amounts of pyridinic N, while using pyrolysis of nitrogen-containing precursors the amount of graphitic N was higher. Using X-ray absorption spectroscopy, the authors were able to identify different nitrogen configurations: three characteristic peaks were found at 399.1, 400.1 and 401.5 eV and were assigned to pyridinic N, cyanide (triple C–N bond) and graphitic N, respectively. This was in agreement with previous reports that correlate XAS and XPS [108–109]. By correlating XAS and RRDE measurements, they assumed that the π* profile observed in the absorption spectra can be used to identify the nitrogen configuration, being therefore an indicator of the ORR activity.

Nagaiah and co-workers obtained similar results on nitrogen-doped CNTs [106]. In their work, post-synthesis heating treatments were used to modify the surface composition, resulting in different nitrogen functionalities. The material with the highest catalytic performance in the ORR was found to be the one with the highest ratio between graphitic N and pyridinic N.

**Figure 9:**
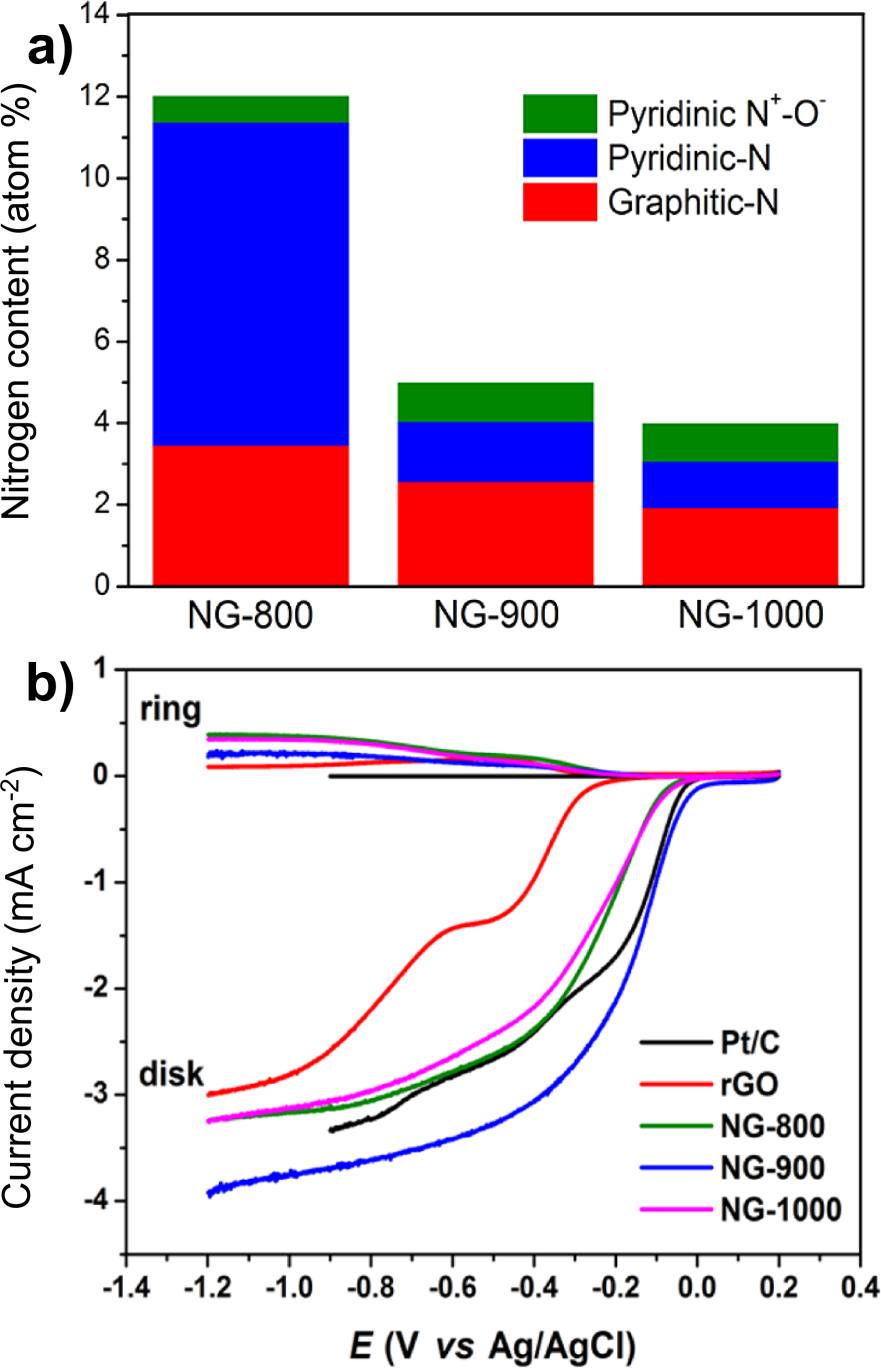
a) Content of the nitrogen species in graphene according to the pyrolysis temperature; b) RRDE voltammetric response for the ORR in O_2_-saturated 0.1 M KOH at a scan rate of 10 mV/s. The electrode rotation rate was 1600 rpm, and the Pt ring electrode was polarized at 0.5 V. Reprinted with permission from [107], copyright 2012 American Chemical Society.

In the report of Parvez et al., nitrogen-doped graphene was synthesized from a composite made of graphitic carbon nitride and graphene sheets, which subsequently underwent heating treatment [107]. Depending on the pyrolysis temperature (800, 900 or 1000 °C), three samples were produced with different nitrogen content and ratio between graphitic N and pyridinic N (Figure 9a). The sample with the highest ratio (1.73) was obtained at 900 °C and this is also the sample with the highest RRDE voltammetric response for the ORR, the only one to surpass the commercial Pt–C catalyst (Figure 9b).

**Figure 10:**
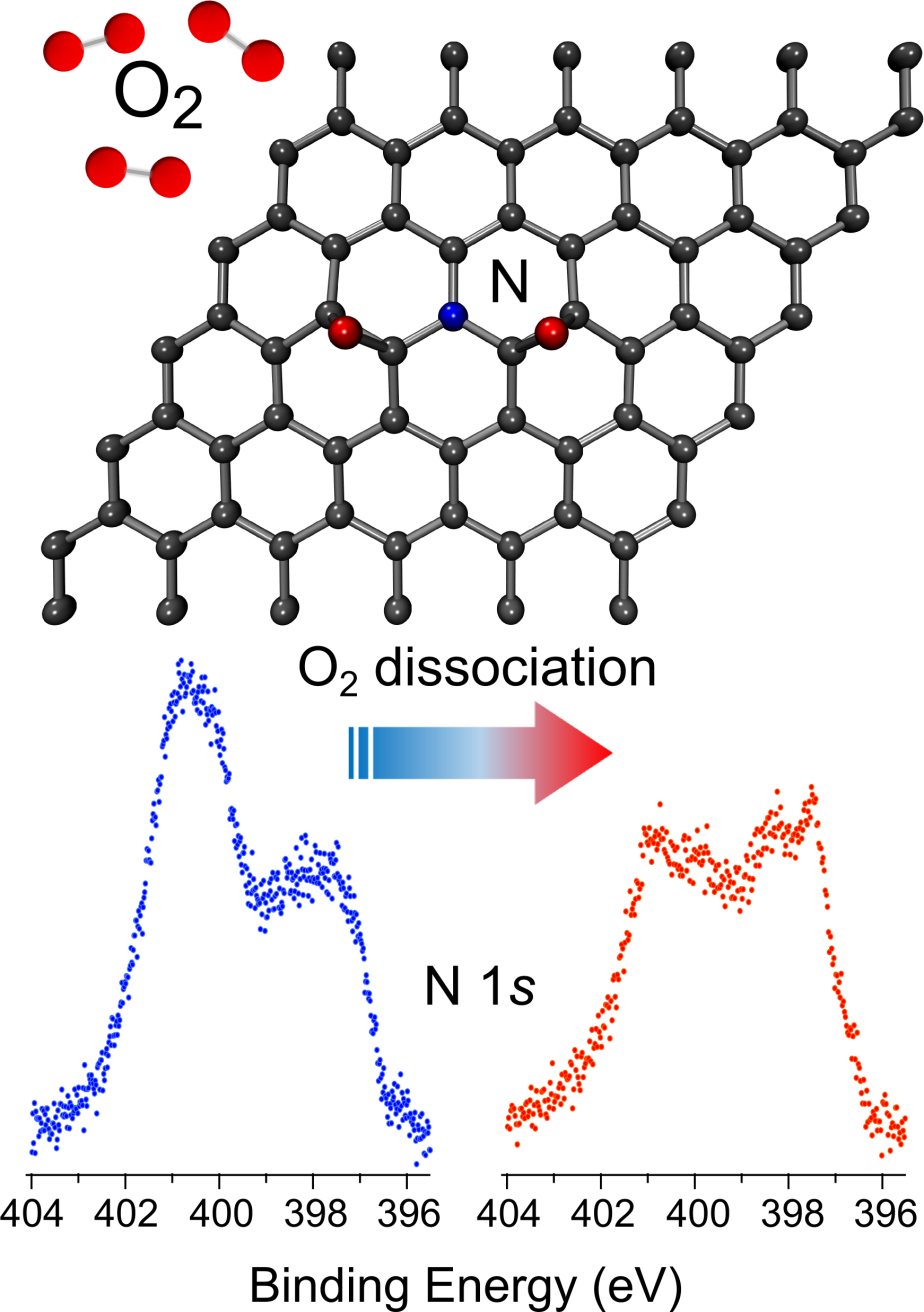
Schematic representation of the molecular oxygen dissociation on nitrogen-doped graphene. Adapted from [84], copyright 2017 the authors, published under a CC-BY 4.0 International license, https://creativecommons.org/licenses/by/4.0/.

Different groups calculated by using DFT the energy barriers for oxygen molecule adsorption and dissociation on pristine and N-doped graphene depending on the nitrogen configuration [21,60,110–112]. Ni and co-workers [111], in particular, found that the energy barrier decreases with all types of nitrogen species, but graphitic nitrogen and Stone–Wales defects are more efficient than pyridinic nitrogen. In fact, the latter was shown to have a negligible occupation of the π*-anti-bonding orbital, due to its two-fold coordination and the lone-pair electrons, in contrast to both graphitic and Stone–Wales defects, which are three-fold coordinated. The dissociation of the oxygen molecule was reported to take place on the carbon atom neighbor of the substitutional nitrogen. The two dissociated O atoms are then adsorbed on an adjacent C–C bridge site, while the C–N bridge site is not available for O adsorption. This mechanism was recently observed by Scardamaglia et al. with high-resolution synchrotron measurements in a fully in situ experiment [84] in which contamination was avoided. The authors functionalized single-layer graphene grown by CVD on iridium(111) using nitrogen plasma and studied its interaction with molecular oxygen. They showed that the interaction results in oxygen dissociation and the formation of carbon–oxygen single bonds on graphene, as observed by X-ray absorption spectroscopy of the carbon K edge and the O 1s core level. After the exposure to molecular oxygen, the N 1s core level spectrum changes its line shape. In particular, the side of higher binding energies (around 401 eV) is quenched. These changes indicate that graphitic nitrogen is involved in the observed mechanism. The adsorbed oxygen molecule is dissociated and the two O atoms chemisorb with epoxy bonds to the nearest carbon neighbors of the graphitic nitrogen atom, as schematized in Figure 10.

**Figure 11:**
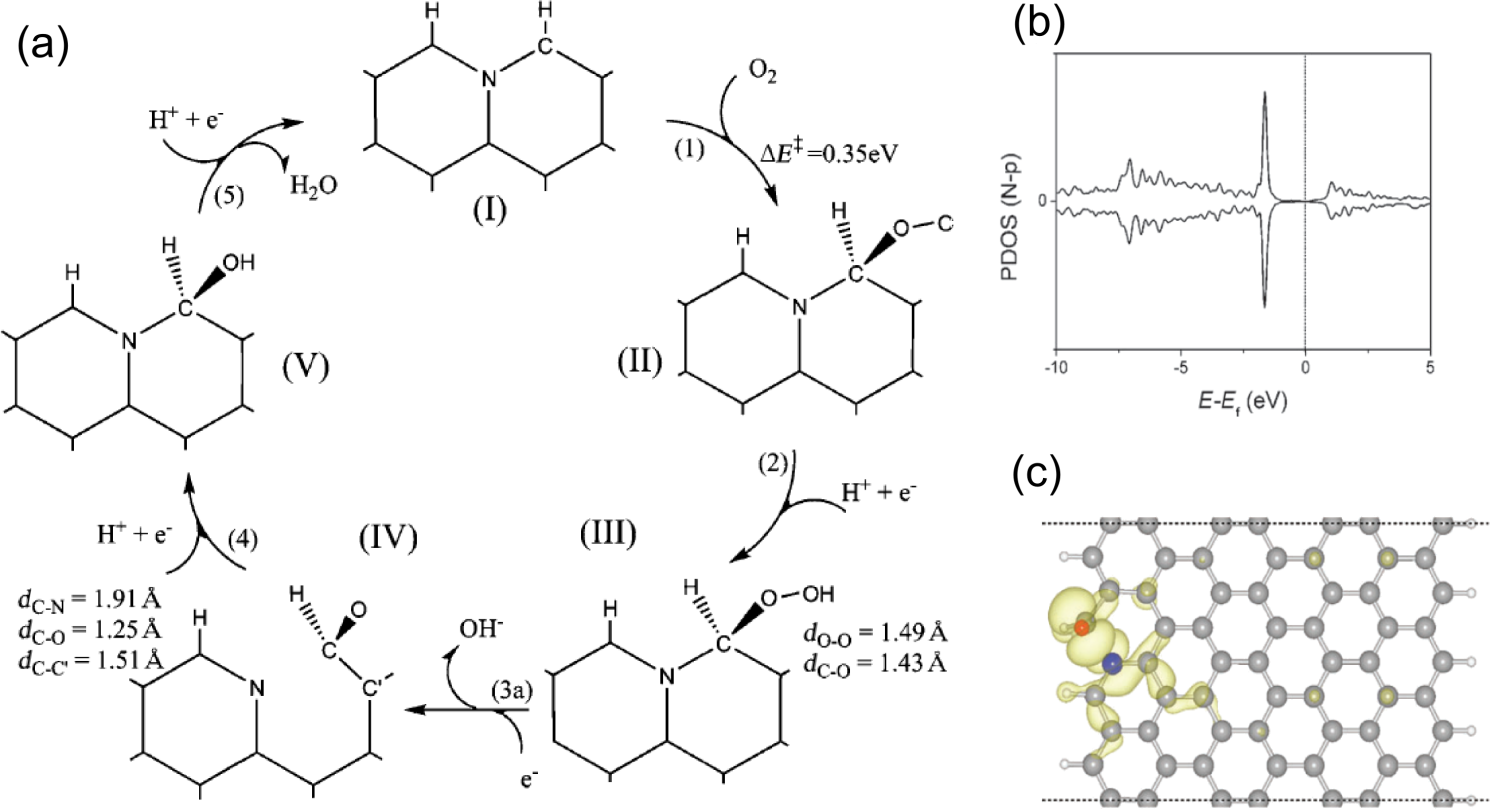
Figure 11 (a) The proposed ORR catalytic cycle. (b) The partial density of states (PDOS) for the p orbital of the nitrogen atom in stage (IV) of (a). (c) The isosurface of electron density for the band of the peak position around −2 eV. The blue and red atoms are nitrogen and oxygen, respectively. Reproduced with permission from [112], copyright 2011 Royal Society of Chemistry.

Theoretical studies on graphene nanoribbons allowed for the distinction of the catalytic activity of nitrogen atoms with different configurations in a graphitic lattice. Because of the higher intrinsic reactivity of the graphene edge sites, oxygen adsorption at those positions has a much lower energy barrier than at “planar bulk” sites, independently from the eventual functionalization [113]. Nitrogen doping increases the activity of the edges and enhances the electron transfer rate because the energy difference between the Fermi level and the unoccupied 2p orbital state in the adsorbed oxygen molecule is reduced. Furthermore, the ORR pathway in the presence of nitrogen is a four-electron reduction process, instead of the less efficient two-electron process in the pristine graphene nanoribbon. Kim et al. compared the catalytic activity of graphitic N localized at different sites with respect to the graphene nanoribbon edge reporting. They found that the catalytically most active sites are those near the edges, the outermost graphitic or graphitic valley N atoms [112]. Their results are in agreement with Ikeda and co-workers who stated that an O_2_ molecule is preferentially adsorbed associatively at C sites on graphene-like zigzag edges if a graphitic N is located nearby [110]. Furthermore, Kim et al. suggested that the graphitic N catalyzes the reaction through a ring-opening of the cyclic C–N bond, which results in the formation of pyridinic N, as shown in Figure 11. In this way, the controversy between the role of graphitic and pyridinic nitrogen in the catalytic process is partially solved.

**Figure 12:**
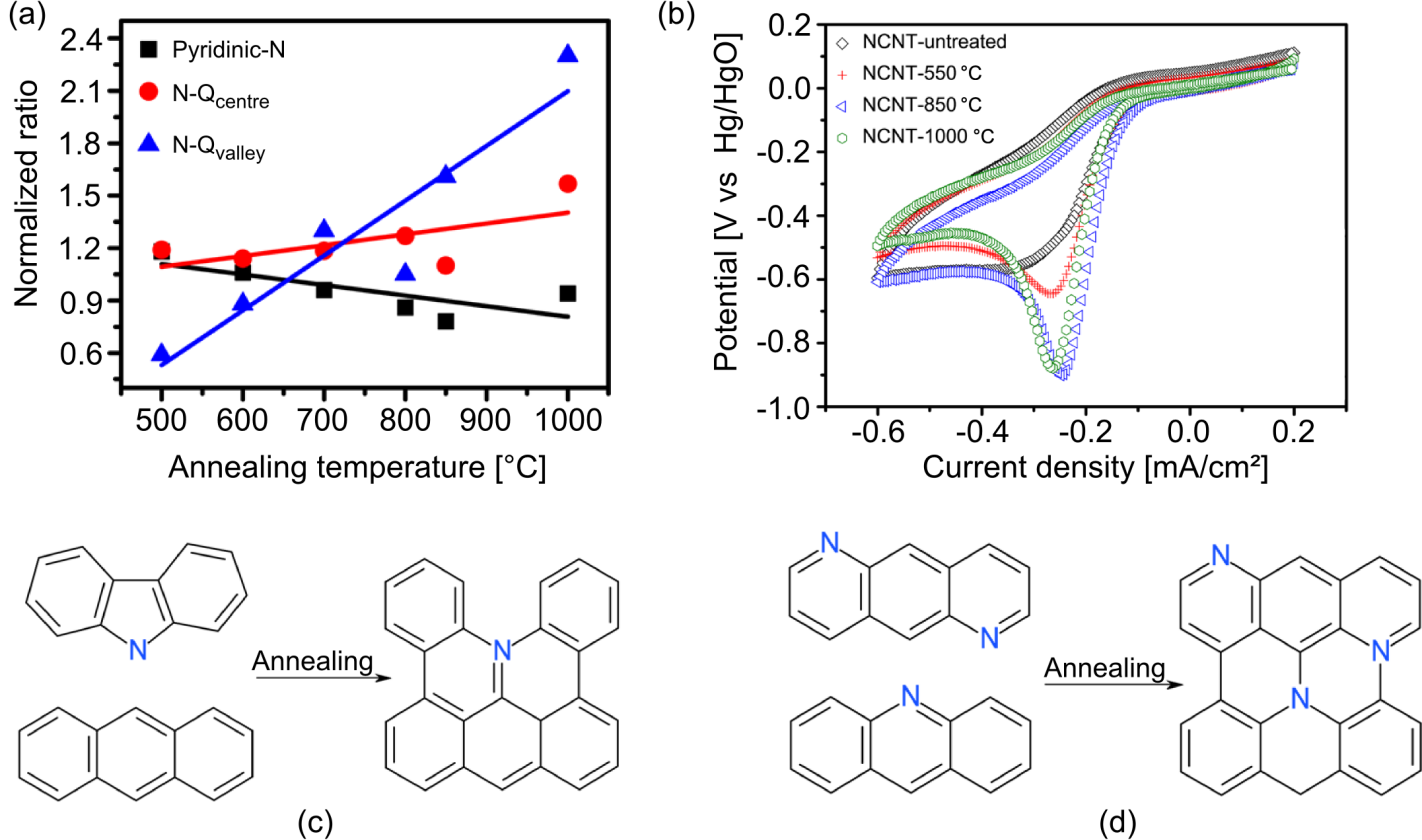
(a) Normalized ratios of nitrogen functionalities as functions of the annealing temperature. (b) Background-corrected cyclic voltammograms in oxygen-saturated 1 M KOH electrolyte for nitrogen-doped CNTs annealed at high temperatures. (c) Condensation reactions by annealing from pyrrolic N to N-Q_valley_ and (d) from pyridinic N to N-Qs (N-Q_center_ and N-Q_valley_). Adapted with permission from [97], copyright 2012 American Chemical Society.

In agreement with the theoretical reports of Ikeda and Kim [110,112], in 2012, Sharifi et al. investigated the formation of different active sites by heat-treatment of nitrogen-doped multiwalled CNTs and subsequent ORR measurements [97]. The nanotubes were synthesized using a pyridine precursor resulting in N contents in the range of 3–6 atom %. However, the starting material already contained a significant amount of oxygen due to the used synthesis conditions, leading to a relatively high concentration of nitrogen oxide groups. Annealing the samples in argon atmosphere at temperatures ranging from 500 to 1000 °C, the authors observed changes induced in the amount and the surface rearrangement of the nitrogen species: a decrease of pyrrolic N and a general transformation into graphitic N, consistent with other reports [58]. The electrocatalytic performances were measured by cyclic voltammetry in oxygen-saturated 1 M KOH electrolyte. By correlating XPS and ORR, the samples with the highest amount of graphitic valley N sites were identified as the most active ones with a four-electron process, as reported in Figure 12, in agreement with theoretical calculations [110,112].

Simultaneously, Lai and co-workers, studied the catalytic activity of two distinct N-doped graphene samples that underwent different temperature treatments, yielding different surface compositions of nitrogen functionalities, as observed by XPS [114]. They produced N-graphene either by the annealing of graphene oxide (GO) in NH_3_ or by annealing a composite of N-containing polymer (polyaniline or polypyrrole) and rGO. In the first case, they obtained mostly graphitic nitrogen and pyridinic nitrogen, in the second one, mostly pyridinic nitrogen and pyrrolic nitrogen. The total nitrogen amount ranged from 3 to 7 atom %. Correlating XPS with electrochemical tests carried out in a standard three-electrode cell with a Pt plate as the counter electrode, they showed that the total N content is not a fundamental parameter in the ORR process. Moreover, graphitic nitrogen and pyridinic nitrogen play different roles. Graphitic nitrogen determines the limiting current density, while pyridinic nitrogen improves the onset potential for the ORR and might convert the ORR reaction mechanism from a two-electron process to a four-electron process.

**Figure 13:**
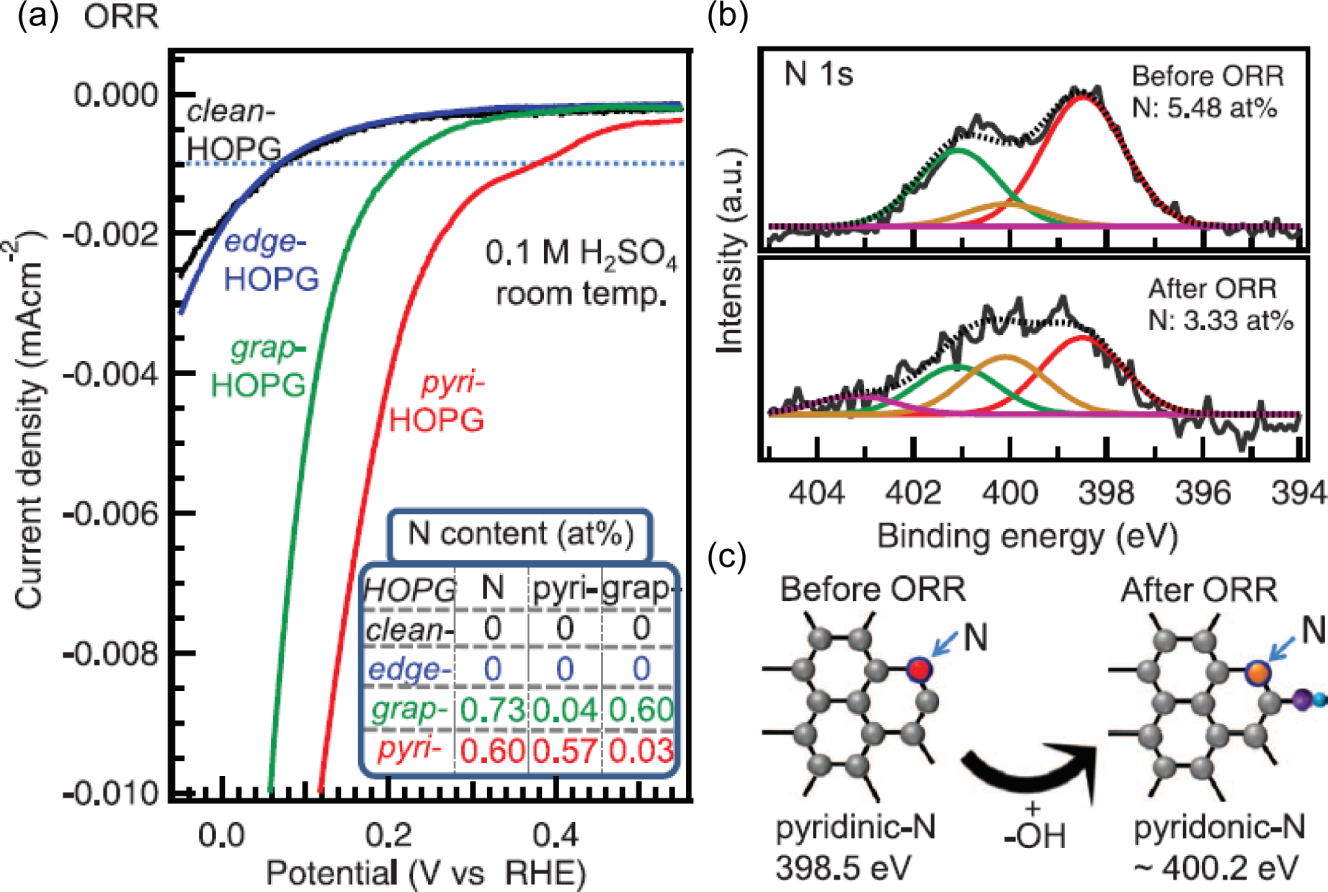
Structural and elemental characterization of four types of N-HOPG model catalysts and their ORR performance. (a) ORR results for model catalysts the nitrogen content of which is reported in the inset. (b,c) Post-ORR XPS analysis of the N-HOPG model catalysts. (b) N 1s XPS spectra of the N-HOPG model catalyst before and after ORR, respectively. (c) Schematic images of the formation of pyridonic N by the attachment of OH to the carbon atom next to pyridinic N. Reprinted with permission from [115], copyright 2016 American Association for the Advancement of Science.

More recently, Guo and co-workers reported a detailed study on highly oriented pyrolytic graphite (HOPG) doped with well-defined N configurations [115]. They achieved the synthesis of model HOPG catalysts with almost only one kind of nitrogen dopant: pyridinic N or graphitic N, with different concentrations up to 11 atom %. By measuring the ORR activity by CV in acidic electrolyte (0.1 M H_2_SO_4_), they demonstrated that the HOPG with pyridinic N is the best catalyst for the ORR, with higher activity at high voltages, and a linear relation between current density and concentration of pyridinic N, without any dependence on the amount of graphitic N (Figure 13a). Performing ex situ XPS after the ORR (Figure 13b), they observed a transformation of the pyridinic configuration into a pyridonic configuration. This is consistent with the adsorption of an O atom on the C atom next to the pyridinic N, as sketched in Figure 13c. Similar results were previously obtained on multilayer graphene by performing XPS measurements after the ORR [116]. This behavior was ascribed to the Lewis basicity of the C atom due to the localized DOS near the Fermi level, as observed by STM [117].

## Conclusion

In all the experiments reported in this review, the picture of the nitrogen active sites in carbon nanomaterials for ORR activity is far from being complete, since many results in similarly designed experiments are contradicting each other. In general, it can be concluded that both nitrogen configurations play a role: graphitic nitrogen determines the limiting current density facilitating the electron transfer from graphene to the antibonding orbitals of the oxygen molecule [104–106,114], while pyridinic nitrogen improves the onset potential for the ORR, by weakening the O–O bond [101–102,115,118]. However, from a theoretical point of view, it has been shown that the activation barrier is much higher for the O_2_ adsorption on both the pyridinic nitrogen site and the nearest carbon atom [60,110–112] than on graphitic nitrogen sites. Likewise, the proximity and the structure of the graphene edge are important in lowering the barrier. This long-lasting debate is primarily due to experimental limitations, namely the low doping level and the lack of control over the nature of the grafted heteroatom functionalities. The heteroatom functionalities strongly depend on the synthesis conditions, causing an inhomogeneous mixing of different configurations of nitrogen atoms. Furthermore, reported experimental studies on the catalytic activities of the active sites in heteroatom-doped carbon nanomaterials are often indirect. An important progress in the field of catalysis is the understanding of the fundamental surface science behind the catalytic process [119]. In particular, from an experimental point of view, recently developed techniques, as (near) ambient-pressure XPS is a very promising tool as it can probe the materials under in operando conditions [120–123]. In this way, it is possible to directly identify the different configurations of the nitrogen atoms in the carbon network using XPS during a chemical reaction. In parallel with the advancement of novel measurement techniques, engineering and synthesis of the catalyst are to be optimized to yield an optimum concentration of active sites without inactive components.
